# Supplementary material for: ProteinShader: illustrative rendering of macromolecules
Source: BMC Struct Biol. 2009 Mar 30;9:19. doi: 10.1186/1472-6807-9-19 (PMC2672931; doi:10.1186/1472-6807-9-19)
Supplement: Additional file 1 — ProteinShader program without source code. This compressed file contains the complete ProteinShader program including associated libraries, but no source code. A README.txt file gives an overview of the ProteinShader distribution, and the index.html file in the help subdirectory has directions on getting started with the program as well as a set of tutorials. [file 1472-6807-9-19-S1.zip › ProteinShader-beta-0_9_4-binary/help/api/org/proteinshader/structure/visitor/enums/AAPortionMode.html]

AAPortionMode (ProteinShader API)


|  |  |  |
| --- | --- | --- |
| **Overview** \| **Package** \| **Class** \| **Use** \| **Tree** \| **Deprecated** \| **Index** \| **Help** | |  |
| PREV CLASS   **NEXT CLASS** | **FRAMES**    **NO FRAMES**     **All Classes** |  |
| SUMMARY: NESTED \| ENUM CONSTANTS \| FIELD \| METHOD | DETAIL: ENUM CONSTANTS \| FIELD \| METHOD |  |


---


## org.proteinshader.structure.visitor.enums Enum AAPortionMode

```
java.lang.Object
  java.lang.Enum<AAPortionMode>
      org.proteinshader.structure.visitor.enums.AAPortionMode
```

**All Implemented Interfaces:**: Serializable, Comparable<AAPortionMode>

---

``` public enum AAPortionMode extends Enum<AAPortionMode> ```

Provides an enumeration that can be used to control what portion of an
AminoAcid the Visitor will traverse.

---

**Enum Constant Summary**

`BACKBONE`
  
          Visit only only the backbone Atoms of an AminoAcid,
'Backbone'.

`C`
  
          Visit only only the carbon Atom of the carbonyl group, 'C'.

`CA`
  
          Visit only only the alpha carbon Atom, 'CA'.

`ENTIRE_AA`
  
          Visit the entire AminoAcid, 'Entire AA'.

`H_OR_HN`
  
          Visit only the hydrogen Atom of the amino group nitrogen,
'H' or "HN'.

`HA_OR_1HA`
  
          Visit only the hydrogen Atom of the alpha carbon, 'HA'
or "1HA'.

`N`
  
          Visit only only the nitrogen Atom of the amino group, 'N'.

`O`
  
          Visit only only the oxygen Atom of the carbonyl group, 'O'.

`OXT`
  
          Visit only only the terminal oxygen Atom of a Chain, 'OXT'.

`SIDE_CHAIN`
  
          Visit only only the side chain Atoms of an AminoAcid,
'Side Chain'.


| **Method Summary** | |
| --- | --- |
| `String` | `getMenuName()`             Returns the name of the AAPortionMode in a form suitable for use in a menu. |
| `String` | `toString()`             Returns the name of the AAPortionMode in a form suitable for use in a menu. |
| `static AAPortionMode` | `valueOf(String name)`             Returns the enum constant of this type with the specified name. |
| `static AAPortionMode` | `valueOfMenuName(String menuName)`             Returns the AAPortionMode with the same menu name as the String given as an argument. |
| `static AAPortionMode[]` | `values()`             Returns an array containing the constants of this enum type, in the order they're declared. |

**Methods inherited from class java.lang.Enum**

`clone, compareTo, equals, getDeclaringClass, hashCode, name, ordinal, valueOf`

**Methods inherited from class java.lang.Object**

`finalize, getClass, notify, notifyAll, wait, wait, wait`

**Enum Constant Detail**

### ENTIRE\_AA

```
public static final AAPortionMode ENTIRE_AA
```

:   Visit the entire AminoAcid, 'Entire AA'.

---


### BACKBONE

```
public static final AAPortionMode BACKBONE
```

:   Visit only only the backbone Atoms of an AminoAcid,
    'Backbone'.

---


### SIDE\_CHAIN

```
public static final AAPortionMode SIDE_CHAIN
```

:   Visit only only the side chain Atoms of an AminoAcid,
    'Side Chain'.

---


### N

```
public static final AAPortionMode N
```

:   Visit only only the nitrogen Atom of the amino group, 'N'.

---


### CA

```
public static final AAPortionMode CA
```

:   Visit only only the alpha carbon Atom, 'CA'.

---


### C

```
public static final AAPortionMode C
```

:   Visit only only the carbon Atom of the carbonyl group, 'C'.

---


### HA\_OR\_1HA

```
public static final AAPortionMode HA_OR_1HA
```

:   Visit only the hydrogen Atom of the alpha carbon, 'HA'
    or "1HA'.

---


### H\_OR\_HN

```
public static final AAPortionMode H_OR_HN
```

:   Visit only the hydrogen Atom of the amino group nitrogen,
    'H' or "HN'.

---


### O

```
public static final AAPortionMode O
```

:   Visit only only the oxygen Atom of the carbonyl group, 'O'.

---


### OXT

```
public static final AAPortionMode OXT
```

:   Visit only only the terminal oxygen Atom of a Chain, 'OXT'.


**Method Detail**

### values

```
public static final AAPortionMode[] values()
```

:   Returns an array containing the constants of this enum type, in
    the order they're declared. This method may be used to iterate
    over the constants as follows:

    ```
    for(AAPortionMode c : AAPortionMode.values())
            System.out.println(c);
    ```

    :   **Returns:**: an array containing the constants of this enum type, in the order they're declared

---


### valueOf

```
public static AAPortionMode valueOf(String name)
```

:   Returns the enum constant of this type with the specified name.
    The string must match *exactly* an identifier used to declare an
    enum constant in this type. (Extraneous whitespace characters are
    not permitted.)

    :   **Parameters:**: `name` - the name of the enum constant to be returned. **Returns:**: the enum constant with the specified name **Throws:**: `IllegalArgumentException` - if this enum type has no constant with the specified name

---


### getMenuName

```
public String getMenuName()
```

:   Returns the name of the AAPortionMode in a form suitable for use
    in a menu.

    :   **Returns:**: The AAPortionMode as a String.

---


### toString

```
public String toString()
```

:   Returns the name of the AAPortionMode in a form suitable for use
    in a menu.

    :   **Overrides:**: `toString` in class `Enum<AAPortionMode>`
    :   **Returns:**: The AAPortionMode as a String.

---


### valueOfMenuName

```
public static AAPortionMode valueOfMenuName(String menuName)
```

:   Returns the AAPortionMode with the same menu name as the String
    given as an argument.

    :   **Returns:**: The AAPortionMode matching the menu name. **Throws:**: `IllegalArgumentException` - if the menu name does not match a AAPortionMode.


---


|  |  |  |
| --- | --- | --- |
| **Overview** \| **Package** \| **Class** \| **Use** \| **Tree** \| **Deprecated** \| **Index** \| **Help** | |  |
| PREV CLASS   **NEXT CLASS** | **FRAMES**    **NO FRAMES**     **All Classes** |  |
| SUMMARY: NESTED \| ENUM CONSTANTS \| FIELD \| METHOD | DETAIL: ENUM CONSTANTS \| FIELD \| METHOD |  |


---

# *Copyright © 2007-2008*
